# Supplementary material for: TIM, a targeted insertional mutagenesis method utilizing CRISPR/Cas9 in Chlamydomonas reinhardtii
Source: PLoS One. 2020 May 13;15(5):e0232594. doi: 10.1371/journal.pone.0232594 (PMC7219734; doi:10.1371/journal.pone.0232594)
Supplement: S1 Appendix — (DOCX) [file pone.0232594.s001.docx]

**Appendix S1. Sequences from *fap70* mutants, donor DNA, and wild type**

>C1

**CGCAGCGCGAAACCCACATC**ACCCTCAACATCCCGGGCTGCCACGTTCCCTGGGCCTCAGCCCACACCGCGCCCTCGGAGGGCGGCGTGTACCAGTACAGCGTGGTGAAGCACTTCAGGCGCTCGGGCGGCCAGGACGGGCTCCTGCAGCTCATCAACGGCGTGTTGACTGTGACCGTGAACGACAGCGCCACCAATGCGGTGCTCGCCAGCGCCACCGTTGACCAGCTGCAGGGGTTTGCAATCGGTCAGAGTAAAACGACGGCCAGTGAATTGTAATACGACTCACTATAGGGCGAATTGGAGCTCTTTCTTGCGCTATGACACTTCCAGCAAAAGGTAGGGCGGGCTGCGAGACGGCTTCCCGGCGCTGCATGCAACACCGATGATGCTTCGACCCCCCGAAGCTCCTTCGGGGCTGCATGGGCGCTCCGATGCCGCTCCAGGGCGAGCGCTGTTTAAATAGCCAGGCCCCCGATTGCAAAGACATTATAGCGAGCTACCAAAGCCATATTCAAACACCTAGATCACTACCACTTCTACACAGGCCACTCGAGCTTGTGATCGCACTCCGCTAAGGGGGCGCCTCTTCCTCTTCGTTTCAGTCACAACCCGCAAACATGGTCGAGATTCGAAGCATGGACGATGCGTTGCGTGCACTGCGGGGTCGGTATCCCGGTTGTGAGTGGGTTGTTGTGGAGGATGGGGCCTCGGGGGCTGGTGTTTATCGGCTTCGGGGTGGTGGGCGGGAGTTGTTTGTCAAGGTGGCAGCTCTGGGGGCCGGGGTGGGCTTGTTGGGTGAGGCTGAGCGGCTGGTGTGGTTGGCGGAGGTGGGGATTCCCGTACCTCGTGTTGTGGAGGGTGGTGGGGACGAGAGGGTCGCCTGGTTGGTCACCGAAGCGGTTCCGGGGCGTCCGGCCAGTGCGCGGTGGCCGCGGGAGCAGCGGCTGGACGTGGCGGTGGCGCTCGCGGGGCTCGCTCGTTCGCTGCACGCGCTGGACTGGGAGCGGTGTCCGTTCGATCGCAGTCTCGCGGTGACGGTGCCGCAGGCGGCCCGTGCTGTCGCTGAAGGGAGCGTCGACTTGGAGGATCTGGACGAGGAGCGGAAGGGGTGGTCGGGGGAGCGGCTTCTCGCCGAGCTGGAGCGGACTCGGCCTGCGGACGAGGATCTGGCGGTTTGCCACGGTGACCTGTGCCCGGACAACGTGCTGCTCGACCCTCGTACCTGCGAGGTGACCGGGCTGATCGACGTGGGGCGGGTCGGCCGTGCGGACCGGCACTCCGATCTCGCGCTGGTGCTGCGCGAGCTGGCCCACGAGGAGGACCCGTGGTTCGGGCCGGAGTGTTCCGCGGCGTTCCTGCGGGAGTACGGGCGCGGGTGGGATGGGGCGGTATCGGAGGAAAAGCTGGCGTTTTACCGGCTGTTGGACGAGTTCTTCTGAGGGACCTGATGGTGTTGGTGGCTGGGTAGGGTTGCGTCGCGTGGGTGACAGCACAGTGTGGACGTTGGGATCCGGCAAGACTGGCCCCGCTTGGCAACGCAACAGTGAGCCCCTCCCTAGTGTGTTTGGGGATGTGACTATGTATTCGTGTGTTGGCCAACGGGTCAACCCGAACAGATTGATACCCGCCTTGGCATTTCCTGTCAGAATGTAACGTCAGTTGATGGTACCAGCTTTTGTTCCCTTTAGTGAGGGTTAATTTCGAGCTTGGCGTAATCATGGTCATAGCTGTTTCCTGTGTGAAATTGTTATCCGCTCACAATTCCACACAACATACGAGCCGGAAGCATAAAGTGTAAAGCCTGGGGTGCCTAATGAGTGAGCTAACTCACATTAATTGCGTTGCGCTCACTGCCCGCTTTCCAGTCGGGAAACCTGTCGTGCCAGCCGCAGCGGAGGTG**CCGGAGGGGGTGCCCAAG**

>C2

**CGCAGCGCGAAACCCACATC**ACCCTCAACATCCCGGGCTGCCACGTTCCCTGGGCCTCAGCCCACACCGCGCCCTCGGAGGGCGGCGTGTACCAGTACAGCGTGGTGAAGCACTTCAGGCGCTCGGGCGGCCAGGACGGGCTCCTGCAGCTCATCAACGGCGTGTTGACTGTGACCGTGAACGACAGCGCCACCAATGCGGTGCTCGCCAGCGCCACCGTTGACCAGCTGCAGGGGTTTGCAATCGGTCAGAGTAAAACGACGGCCAGTGAATTGTAATACGACTCACTATAGGGCGAATTGGAGCTCTTTCTTGCGCTATGACACTTCCAGCAAAAGGTAGGGCGGGCTGCGAGACGGCTTCCCGGCGCTGCATGCAACACCGATGATGCTTCGACCCCCCGAAGCTCCTTCGGGGCTGCATGGGCGCTCCGATGCCGCTCCAGGGCGAGCGCTGTTTAAATAGCCAGGCCCCCGATTGCAAAGACATTATAGCGAGCTACCAAAGCCATATTCAAACACCTAGATCACTACCACTTCTACACAGGCCACTCGAGCTTGTGATCGCACTCCGCTAAGGGGGCGCCTCTTCCTCTTCGTTTCAGTCACAACCCGCAAACATGGTCGAGATTCGAAGCATGGACGATGCGTTGCGTGCACTGCGGGGTCGGTATCCCGGTTGTGAGTGGGTTGTTGTGGAGGATGGGGCCTCGGGGGCTGGTGTTTATCGGCTTCGGGGTGGTGGGCGGGAGTTGTTTGTCAAGGTGGCAGCTCTGGGGGCCGGGGTGGGCTTGTTGGGTGAGGCTGAGCGGCTGGTGTGGTTGGCGGAGGTGGGGATTCCCGTACCTCGTGTTGTGGAGGGTGGTGGGGACGAGAGGGTCGCCTGGTTGGTCACCGAAGCGGTTCCGGGGCGTCCGGCCAGTGCGCGGTGGCCGCGGGAGCAGCGGCTGGACGTGGCGGTGGCGCTCGCGGGGCTCGCTCGTTCGCTGCACGCGCTGGACTGGGAGCGGTGTCCGTTCGATCGCAGTCTCGCGGTGACGGTGCCGCAGGCGGCCCGTGCTGTCGCTGAAGGGAGCGTCGACTTGGAGGATCTGGACGAGGAGCGGAAGGGGTGGTCGGGGGAGCGGCTTCTCGCCGAGCTGGAGCGGACTCGGCCTGCGGACGAGGATCTGGCGGTTTGCCACGGTGACCTGTGCCCGGACAACGTGCTGCTCGACCCTCGTACCTGCGAGGTGACCGGGCTGATCGACGTGGGGCGGGTCGGCCGTGCGGACCGGCACTCCGATCTCGCGCTGGTGCTGCGCGAGCTGGCCCACGAGGAGGACCCGTGGTTCGGGCCGGAGTGTTCCGCGGCGTTCCTGCGGGAGTACGGGCGCGGGTGGGATGGGGCGGTATCGGAGGAAAAGCTGGCGTTTTACCGGCTGTTGGACGAGTTCTTCTGAGGGACCTGATGGTGTTGGTGGCTGGGTAGGGTTGCGTCGCGTGGGTGACAGCACAGTGTGGACGTTGGGATCCGGCAAGACTGGCCCCGCTTGGCAACGCAACAGTGAGCCCCTCCCTAGTGTGTTTGGGGATGTGACTATGTATTCGTGTGTTGGCCAACGGGTCAACCCGAACAGATTGATACCCGCCTTGGCATTTCCTGTCAGAATGTAACGTCAGTTGATGGTACCAGCTTTTGTTCCCTTTAGTGAGGGTTAATTTCGAGCTTGGCGTAATCATGGTCATAGCTGTTTCCTGTGTGAAATTGTTATCCGCTCACAATTCCACACAACATACGAGCCGGAAGCATAAAGTGTAAAGCCTGGGGTGCCTAATGAGTGAGCTAACTCACATTAATTGCGTTGCGCTCACTGCCCGCTTTCCAGTCGGGAAACCTGTCGTGCCAGCCGCAGCGGAGGTG**CCGGAGGGGGTGCCCAAG**

>C3

**CGCAGCGCGAAACCCACATC**ACCCTCAACATCCCGGGCTGCCACGTTCCCTGGGCCTCAGCCCACACCGCGCCCTCGGAGGGCGGCGTGTACCAGTACAGCGTGGTGAAGCACTTCAGGCGCTCGGGCGGCCAGGACGGGCTCCTGCAGCTCATCAACGGCGTGTTGACTGTGACCGTGAACGACAGCGCCACCAATGCGGTGCTCGCCAGCGCCACCGTTGACCAGCTGCAGGGGTTTGCAATCGGTCAGAGTAAAACGACGGCCAGTGAATTGTAATACGACTCACTATAGGGCGAATTGGAGCTCTTTCTTGCGCTATGACACTTCCAGCAAAAGGTAGGGCGGGCTGCGAGACGGCTTCCCGGCGCTGCATGCAACACCGATGATGCTTCGACCCCCCGAAGCTCCTTCGGGGCTGCATGGGCGCTCCGATGCCGCTCCAGGGCGAGCGCTGTTTAAATAGCCAGGCCCCCGATTGCAAAGACATTATAGCGAGCTACCAAAGCCATATTCAAACACCTAGATCACTACCACTTCTACACAGGCCACTCGAGCTTGTGATCGCACTCCGCTAAGGGGGCGCCTCTTCCTCTTCGTTTCAGTCACAACCCGCAAACATGGTCGAGATTCGAAGCATGGACGATGCGTTGCGTGCACTGCGGGGTCGGTATCCCGGTTGTGAGTGGGTTGTTGTGGAGGATGGGGCCTCGGGGGCTGGTGTTTATCGGCTTCGGGGTGGTGGGCGGGAGTTGTTTGTCAAGGTGGCAGCTCTGGGGGCCGGGGTGGGCTTGTTGGGTGAGGCTGAGCGGCTGGTGTGGTTGGCGGAGGTGGGGATTCCCGTACCTCGTGTTGTGGAGGGTGGTGGGGACGAGAGGGTCGCCTGGTTGGTCACCGAAGCGGTTCCGGGGCGTCCGGCCAGTGCGCGGTGGCCGCGGGAGCAGCGGCTGGACGTGGCGGTGGCGCTCGCGGGGCTCGCTCGTTCGCTGCACGCGCTGGACTGGGAGCGGTGTCCGTTCGATCGCAGTCTCGCGGTGACGGTGCCGCAGGCGGCCCGTGCTGTCGCTGAAGGGAGCGTCGACTTGGAGGATCTGGACGAGGAGCGGAAGGGGTGGTCGGGGGAGCGGCTTCTCGCCGAGCTGGAGCGGACTCGGCCTGCGGACGAGGATCTGGCGGTTTGCCACGGTGACCTGTGCCCGGACAACGTGCTGCTCGACCCTCGTACCTGCGAGGTGACCGGGCTGATCGACGTGGGGCGGGTCGGCCGTGCGGACCGGCACTCCGATCTCGCGCTGGTGCTGCGCGAGCTGGCCCACGAGGAGGACCCGTGGTTCGGGCCGGAGTGTTCCGCGGCGTTCCTGCGGGAGTACGGGCGCGGGTGGGATGGGGCGGTATCGGAGGAAAAGCTGGCGTTTTACCGGCTGTTGGACGAGTTCTTCTGAGGGACCTGATGGTGTTGGTGGCTGGGTAGGGTTGCGTCGCGTGGGTGACAGCACAGTGTGGACGTTGGGATCCGGCAAGACTGGCCCCGCTTGGCAACGCAACAGTGAGCCCCTCCCTAGTGTGTTTGGGGATGTGACTATGTATTCGTGTGTTGGCCAACGGGTCAACCCGAACAGATTGATACCCGCCTTGGCATTTCCTGTCAGAATGTAACGTCAGTTGATGGTACCAGCTTTTGTTCCCTTTAGTGAGGGTTAATTTCGAGCTTGGCGTAATCATGGTCATAGCTGTTTCCTGTGTGAAATTGTTATCCGCTCACAATTCCACACAACATACGAGCCGGAAGCATAAAGTGTAAAGCCTGGGGTGCCTAATGAGTGAGCTAACTCACATTAATTGCGTTGCGCTCACTGCCCGCTTTCCAGTCGGGAAACCTGTCGTGCCAGCCGCAGCGGAGGTG**CCGGAGGGGGTGCCCAAG**

>C6

**CGCAGCGCGAAACCCACATC**ACCCTCAACATCCCGGGCTGCCACGTTCCCTGGGCCTCAGCCCACACCGCGCCCTCGGAGGGCGGCGTGTACCAGTACAGCGTGGTGAAGCACTTCAGGCGCTCGGGCGGCCAGGACGGGCTCCTGCAGCTCATCAACGGCGTGTTGACTGTGACCGTGAACGACAGCGCCACCAATGCGGTGCTCGCCAGCGCCACCGTTGACCAGCTGCAGGGGTTTGCAATCGGTCAGAACACATGGAGCACCGAGGGTCTCGACGCATGGGCGCTCCGATGCCGCTCCAGGGCGAGCGCTGTTTAAATAGCCAGGCCCCCGATTGCAAAGACATTATAGCGAGCTACCAAAGCCATATTCAAACACCTAGATCACTACCACTTCTACACAGGCCACTCGAGCTTGTGATCGCACTCCGCTAAGGGGGCGCCTCTTCCTCTTCGTTTCAGTCACAACCCGCAAACATGGTCGAGATTCGAAGCATGGACGATGCGTTGCGTGCACTGCGGGGTCGGTATCCCGGTTGTGAGTGGGTTGTTGTGGAGGATGGGGCCTCGGGGGCTGGTGTTTATCGGCTTCGGGGTGGTGGGCGGGAGTTGTTTGTCAAGGTGGCAGCTCTGGGGGCCGGGGTGGGCTTGTTGGGTGAGGCTGAGCGGCTGGTGTGGTTGGCGGAGGTGGGGATTCCCGTACCTCGTGTTGTGGAGGGTGGTGGGGACGAGAGGGTCGCCTGGTTGGTCACCGAAGCGGTTCCGGGGCGTCCGGCCAGTGCGCGGTGGCCGCGGGAGCAGCGGCTGGACGTGGCGGTGGCGCTCGCGGGGCTCGCTCGTTCGCTGCACGCGCTGGACTGGGAGCGGTGTCCGTTCGATCGCAGTCTCGCGGTGACGGTGCCGCAGGCGGCCCGTGCTGTCGCTGAAGGGAGCGTCGACTTGGAGGATCTGGACGAGGAGCGGAAGGGGTGGTCGGGGGAGCGGCTTCTCGCCGAGCTGGAGCGGACTCGGCCTGCGGACGAGGATCTGGCGGTTTGCCACGGTGACCTGTGCCCGGACAACGTGCTGCTCGACCCTCGTACCTGCGAGGTGACCGGGCTGATCGACGTGGGGCGGGTCGGCCGTGCGGACCGGCACTCCGATCTCGCGCTGGTGCTGCGCGAGCTGGCCCACGAGGAGGACCCGTGGTTCGGGCCGGAGTGTTCCGCGGCGTTCCTGCGGGAGTACGGGCGCGGGTGGGATGGGGCGGTATCGGAGGAAAAGCTGGCGTTTTACCGGCTGTTGGACGAGTTCTTCTGAGGGACCTGATGGTGTTGGTGGCTGGGTAGGGTTGCGTCGCGTGGGTGACAGCACAGTGTGGACGTTGGGATCCGGCAAGACTGGCCCCGCTTGGCAACGCAACAGTGAGCCCCTCCCTAGTGTGTTTGGGGATGTGACTATGTATTCGTGTGTTGGCCAACGGGTCAACCCGAACAGATTGATACCCGCCTTGGCATTTCCTGTCAGAATGTAACGTCAGTTGATGGTACCAGCTTTTGTTCCCTTTAGTGAGGGTTAATTTCGAGCTTGGCGTAATCATGGGCGGAGCGACCCATGCATCTGGTGCCCGCAGCGGAGGTG**CCGGAGGGGGTGCCCAAG**

>D1

**CGCAGCGCGAAACCCACATC**ACCCTCAACATCCCGGGCTGCCACGTTCCCTGGGCCTCAGCCCACACCGCGCCCTCGGAGGGCGGCGTGTACCAGTACAGCGTGGTGAAGCACTTCAGGCGCTCGGGCGGCCAGGACGGGCTCCTGCAGCTCATCAACGGCGTGTTGACTGTGACCGTGAACGACAGCGCCACCAATGCGGTGCTCGCCAGCGCCACCGTTGACCAGCTGCAGGGGTTTGCAATCGGTCAGAGTAAAACGACGGCCAGTGAATTGTAATACGACTCACTATAGGGCGAATTGGAGCTCTTTCTTGCGCTATGACACTTCCAGCAAAAGGTAGGGCGGGCTGCGAGACGGCTTCCCGGCGCTGCATGCAACACCGATGATGCTTCGACCCCCCGAAGCTCCTTCGGGGCTGCATGGGCGCTCCGATGCCGCTCCAGGGCGAGCGCTGTTTAAATAGCCAGGCCCCCGATTGCAAAGACATTATAGCGAGCTACCAAAGCCATATTCAAACACCTAGATCACTACCACTTCTACACAGGCCACTCGAGCTTGTGATCGCACTCCGCTAAGGGGGCGCCTCTTCCTCTTCGTTTCAGTCACAACCCGCAAACATGGTCGAGATTCGAAGCATGGACGATGCGTTGCGTGCACTGCGGGGTCGGTATCCCGGTTGTGAGTGGGTTGTTGTGGAGGATGGGGCCTCGGGGGCTGGTGTTTATCGGCTTCGGGGTGGTGGGCGGGAGTTGTTTGTCAAGGTGGCAGCTCTGGGGGCAGGGGTGGGCTTGTTGGGTGAGGCTGAGCGGCTGGTGTGGTTGGCGGAGGTGGGGATTCCCGTACCTCGTGTTGTGGAGGGTGGTGGGGACGAGAGGGTCGCCTGGTTGGTCACCGAAGCGGTTCCGGGGCGTCCGGCCAGTGCGCGGTGGCCGCGGGAGCAGCGGCTGGACGTGGCGGTGGCGCTCGCGGGGCTCGCTCGTTCGCTGCACGCGCTGGACTGGGAGCGGTGTCCGTTCGATCGCAGTCTCGCGGTGACGGTGCCGCAGGCGGCCCGTGCTGTCGCTGAAGGGAGCGTCGACTTGGAGGATCTGGACGAGGAGCGGAAGGGGTGGTCGGGGGAGCGGCTTCTCGCCGAGCTGGAGCGGACTCGGCCTGCGGACGAGGATCTGGCGGTTTGCCACGGTGACCTGTGCCCGGACAACGTGCTGCTCGACCCTCGTACCTGCGAGGTGACCGGGCTGATCGACGTGGGGCGGGTCGGCCGTGCGGACCGGCACTCCGATCTCGCGCTGGTGCTGCGCGAGCTGGCCCACGAGGAGGACCCGTGGTTCGGGCCGGAGTGTTCCGCGGCGTTCCTGCGGGAGTACGGGCGCGGGTGGGATGGGGCGGTATCGGAGGAAAAGCTGGCGTTTTACCGGCTGTTGGACGAGTTCTTCTGAGGGACCTGATGGTGTTGGTGGCTGGGTAGGGTTGCGTCGCGTGGGTGACAGCACAGTGTGGACGTTGGGATCCGGCAAGACTGGCCCCGCTTGGCAACGCAACAGTGAGCCCCTCCCTAGTGTGTTTGGGGATGTGACTATGTATTCGTGTGTTGGCCAACGGGTCAACCCGAACAGATTGATACCCGCCTTGGCATTTCCTGTCAGAATGTAACGTCAGTTGATGGTACCAGCTTTTGTTCCCTTTAGTGAGGGTTAATTTCGAGCTTGGCGTAATCATGGTCATAGCTGTTTCCTGTGTGAAATTGTTATCCGCTCACAATTCCACACAACATACGAGCCGGAAGCATAAAGTGTAAAGCCTGGGGTGCCTAATGAGTGAGCTAACTCACATTAATTGCGTTGCGCTCACTGCCCGCTTTCCAGTCGGGAAACCTGTCGTGCCAGCCGCAGCGGAGGTG**CCGGAGGGGGTGCCCAAG**

>D4

**CGCAGCGCGAAACCCACATC**ACCCTCAACATCCCGGGCTGCCACGTTCCCTGGGCCTCAGCCCACACCGCGCCCTCGGAGGGCGGCGTGTACCAGTACAGCGTGGTGAAGCACTTCAGGCGCTCGGGCGGCCAGGACGGGCTCCTGCAGCTCATCAACGGCGTGTTGACTGTGACCGTGAACGACAGCGCCACCAATGCGGTGCTCGCCAGCGCCACCGTTGACCAGCTGCAGGGGTTTGCAATCGGTCAGAGTAAAACGACGGCCAGTGAATTGTAATACGACTCACTATAGGGCGAATTGGAGCTCTTTCTTGCGCTATGACACTTCCAGCAAAAGGTAGGGCGGGCTGCGAGACGGCTTCCCGGCGCTGCATGCAACACCGATGATGCTTCGACCCCCCGAAGCTCCTTCGGGGCTGCATGGGCGCTCCGATGCCGCTCCAGGGCGAGCGCTGTTTAAATAGCCAGGCCCCCGATTGCAAAGACATTATAGCGAGCTACCAAAGCCATATTCAAACACCTAGATCACTACCACTTCTACACAGGCCACTCGAGCTTGTGATCGCACTCCGCTAAGGGGGCGCCTCTTCCTCTTCGTTTCAGTCACAACCCGCAAACATGGTCGAGATTCGAAGCATGGACGATGCGTTGCGTGCACTGCGGGGTCGGTATCCCGGTTGTGAGTGGGTTGTTGTGGAGGATGGGGCCTCGGGGGCTGGTGTTTATCGGCTTCGGGGTGGTGGGCGGGAGTTGTTTGTCAAGGTGGCAGCTCTGGGGGCCGGGGTGGGCTTGTTGGGTGAGGCTGAGCGGCTGGTGTGGTTGGCGGAGGTGGGGATTCCCGTACCTCGTGTTGTGGAGGGTGGTGGGGACGAGAGGGTCGCCTGGtTGGTCACCGAAGCGGTTCCGGGGCGTCCGGCCAGTGCGCGGtGGCCGCGGGAGCAGCGGCTGGaCGTGGCGGTGGCGCTCGCGGGGCTCGCTCGTTCGCTGCACGCGCTGGACTGGGAGCGGTGTCCGTTCGATCGCAGTCTCGCGGTGACGGTGCCGCAGGCGGCCCGTGCTGTCGCTGAAGGGAGCGTCGACTTGGAGGATCTGGACGAGGAGCGGAAGGGGTGGTCGGGGGAGCGGCTTCTCGCCGAGCTGGAGCGGACTCGGCCTGCGGACGAGGATCTGGCGGTTTGCCACGGTGACCTGTGCCCGGACAACGTGCTGCTCGACCCTCGTACCTGCGAGGTGACCGGGCTGATCGACGTGGGGCGGGTCGGCCGTGCGGACCGGCACTCCGATCTCGCGCTGGTGCTGCGCGAGCTGGCCCACGAGGAGGACCCGTGGTTCGGGCCGGAGTGTTCCGCGGCGTTCCTGCGGGAGTACGGGCGCGGGTGGGATGGGGCGGTATCGGAGGAAAAGCTGGCGTTTTACCGGCTGTTGGACGAGTTCTTCTGAGGGACCTGATGGTGTTGGTGGCTGGGTAGGGTTGCGTCGCGTGGGTGACAGCACAGTGTGGACGTTGGGATCCGGCAAGACTGGCCCCGCTTGGCAACGCAACAGTGAGCCCCTCCCTAGTGTGTTTGGGGATGTGACTATGTATTCGTGTGTTGGCCAACGGGTCAACCCGAACAGATTGATACCCGCCTTGGCATTTCCTGTCAGAATGTAACGTCAGTTGATGGTACCAGCTTTTGTTCCCTTTAGTGAGGGTTAATTTCGAGCTTGGCGTAATCATGGTCATAGCTGTTTCCTGTGTGAAATTGTTATCCGCTCACAATTCCACACAACATACGAGCCGGAAGCATAAAGTGTAAAGCCTGGGGTGCCTAATGAGTGAGCTAACTCACATTAATTGCGTTGCGCTCACTGCCCGCTTTCCAGTCGGGAAACCTGTCGTGCCAGCCGCAGCGGAGGTG**CCGGAGGGGGTGCCCAAG**

>D6

**CGCAGCGCGAAACCCACATC**ACCCTCAACATCCCGGGCTGCCACGTTCCCTGGGCCTCAGCCCACACCGCGCCCTCGGAGGGCGGCGTGTACCAGTACAGCGTGGTGAAGCACTTCAGGCGCTCGGGCGGCCAGGACGGGCTCCTGCAGCTCATCAACGGCGTGTTGACTGTGACCGTGAACGACAGCGCCACCAATGCGGTGCTCGCCAGCGCCACCGTTGACCAGCTGCAGGGGTTTGCAATCGGTCAGAACACATGGAGCACCGAGGGTTAATTTCGAGCTTGGCGTAATCATGGTCATAGCTGTTTCCTGTGTGAAATTGTTATCCGCTCACAATTCCACACAACATACGAGCCGGAAGCATAAAGTGTAAAGCCTGGGGTGCCTAATGAGTGAGCTAACTCACATTAATTGCGTTGCGCTCACTGTGGCTGCATGGGCGCTCCGATGCCGCTCCAGGGCGAGCGCTGTTTAACTAGCCAGGCCCCCGATTGCAAAGACATTATAGCGAGCTACCAAAGCCATATTCAAACACCTAGATCACTACCACTTCTACACAGGCCACTCGAGCTTGTGATCGCACTCCGCTAAGGGGGCGCCTCTTCCTCTTCGTTTCAGTCACAACCCGCAAACATGGTCGAGATTCGAAGCATGGACGATGCGTTGCGTGCACTGCGGGGTCGGTATCCCGGTTGTGAGTGGGTTGTTGTGGAGGATGGGGCCTCGGGGGCTGGTGTTTATCGGCTTCGGGGTGGTGGGCGGGAGTTGTTTGTCAAGGTGGCAGCTCTGGGGGCCGGGGTGGGCTTGTTGGGTGAGGCTGAGCGGCTGGTGTGGTTGGCGGAGGTGGGGATTCCCGTACCTCGTGTTGTGGAGGGTGGTGGGGACGAGAGGGTCGCCTGGTTGGTCACCGAAGCGGTTCCGGGGCGTCCGGCCAGTGCGCGGTGGCCGCGGGAGCAGCGGCTGGACGTGGCGGTGGCGCTCGCGGGGCTCGCTCGTTCGCTGCACGCGCTGGACTGGGAGCGGTGTCCGTTCGATCGCAGTCTCGCGGTGACGGTGCCGCAGGCGGCCCGTGCTGTCGCTGAAGGGAGCGTCGACTTGGAGGATCTGGACGAGGAGCGGAAGGGGTGGTCGGGGGAGCGGCTTCTCGCCGAGCTGGAGCGGACTCGGCCTGCGGACGAGGATCTGGCGGTTTGCCACGGTGACCTGTGCCCGGACAACGTGCTGCTCGACCCTCGTACCTGCGAGGTGACCGGGCTGATCGACGTGGGGCGGGTCGGCCGTGCGGACCGGCACTCCGATCTCGCGCTGGTGCTGCGCGAGCTGGCCCACGAGGAGGACCCGTGGTTCGGGCCGGAGTGTTCCGCGGCGTTCCTGCGGGAGTACGGGCGCGGGTGGGATGGGGCGGTATCGGAGGAAAAGCTGGCGTTTTACCGGCTGTTGGACGAGTTCTTCTGAGGGACCTGATGGTGTTGGTGGCTGGGTAGGGTTGCGTCGCGTGGGTGACAGCACAGTGTGGACGTTGGGATCCGGCAAGACTGGCCCCGCTTGGCAACGCAACAGTGAGCCCCTCCCTAGTGTGTTTGGGGATGTGACTATGTATTCGTGTGTTGGCCAACGGGTCAACCCGAACAGATTGATACCCGCCTTGGCATTTCCTGTCAGAATGTAACGTCAGTTGATGGTACCAGCTTTTGTTCCCTTTAGTGAGGGTTAATTTCGAGCTTGGCGTAATCATGGTCATAGCTGTTTCCTGTGTGAAATTGTTATCCGCTCACAATTCCACACAACATACGAGCCGGAAGCATAAAGTGTAAAGCCTGGGGTGCCTAATGAGTGAGCTAACTCACATTAATTGCGTTGCGCTCACTGCCCGCTTTCCAGTCGGGAAACCTGTCGTGCCAGCCGCAGCGGAGGTG**CCGGAGGGGGTGCCCAAG**

>Donor DNA (paromomycin cassette with 50-bp *FAP70* homology arms)

GCTCGCCAGCGCCACCGTTGACCAGCTGCAGGGGTTTGCAATCGGTCAGAGTAAAACGACGGCCAGTGAATTGTAATACGACTCACTATAGGGCGAATTGGAGCTCTTTCTTGCGCTATGACACTTCCAGCAAAAGGTAGGGCGGGCTGCGAGACGGCTTCCCGGCGCTGCATGCAACACCGATGATGCTTCGACCCCCCGAAGCTCCTTCGGGGCTGCATGGGCGCTCCGATGCCGCTCCAGGGCGAGCGCTGTTTAAATAGCCAGGCCCCCGATTGCAAAGACATTATAGCGAGCTACCAAAGCCATATTCAAACACCTAGATCACTACCACTTCTACACAGGCCACTCGAGCTTGTGATCGCACTCCGCTAAGGGGGCGCCTCTTCCTCTTCGTTTCAGTCACAACCCGCAAACATGGTCGAGATTCGAAGCATGGACGATGCGTTGCGTGCACTGCGGGGTCGGTATCCCGGTTGTGAGTGGGTTGTTGTGGAGGATGGGGCCTCGGGGGCTGGTGTTTATCGGCTTCGGGGTGGTGGGCGGGAGTTGTTTGTCAAGGTGGCAGCTCTGGGGGCCGGGGTGGGCTTGTTGGGTGAGGCTGAGCGGCTGGTGTGGTTGGCGGAGGTGGGGATTCCCGTACCTCGTGTTGTGGAGGGTGGTGGGGACGAGAGGGTCGCCTGGTTGGTCACCGAAGCGGTTCCGGGGCGTCCGGCCAGTGCGCGGTGGCCGCGGGAGCAGCGGCTGGACGTGGCGGTGGCGCTCGCGGGGCTCGCTCGTTCGCTGCACGCGCTGGACTGGGAGCGGTGTCCGTTCGATCGCAGTCTCGCGGTGACGGTGCCGCAGGCGGCCCGTGCTGTCGCTGAAGGGAGCGTCGACTTGGAGGATCTGGACGAGGAGCGGAAGGGGTGGTCGGGGGAGCGGCTTCTCGCCGAGCTGGAGCGGACTCGGCCTGCGGACGAGGATCTGGCGGTTTGCCACGGTGACCTGTGCCCGGACAACGTGCTGCTCGACCCTCGTACCTGCGAGGTGACCGGGCTGATCGACGTGGGGCGGGTCGGCCGTGCGGACCGGCACTCCGATCTCGCGCTGGTGCTGCGCGAGCTGGCCCACGAGGAGGACCCGTGGTTCGGGCCGGAGTGTTCCGCGGCGTTCCTGCGGGAGTACGGGCGCGGGTGGGATGGGGCGGTATCGGAGGAAAAGCTGGCGTTTTACCGGCTGTTGGACGAGTTCTTCTGAGGGACCTGATGGTGTTGGTGGCTGGGTAGGGTTGCGTCGCGTGGGTGACAGCACAGTGTGGACGTTGGGATCCGGCAAGACTGGCCCCGCTTGGCAACGCAACAGTGAGCCCCTCCCTAGTGTGTTTGGGGATGTGACTATGTATTCGTGTGTTGGCCAACGGGTCAACCCGAACAGATTGATACCCGCCTTGGCATTTCCTGTCAGAATGTAACGTCAGTTGATGGTACCAGCTTTTGTTCCCTTTAGTGAGGGTTAATTTCGAGCTTGGCGTAATCATGGTCATAGCTGTTTCCTGTGTGAAATTGTTATCCGCTCACAATTCCACACAACATACGAGCCGGAAGCATAAAGTGTAAAGCCTGGGGTGCCTAATGAGTGAGCTAACTCACATTAATTGCGTTGCGCTCACTGCCCGCTTTCCAGTCGGGAAACCTGTCGTGCCAGCCGCAGCGGAGGTG**CCGGAGGGGGTGCCCAAG**GTGCGCGCGCCGGCCGCT

>Wild type

**CGCAGCGCGAAACCCACATC**ACCCTCAACATCCCGGGCTGCCACGTTCCCTGGGCCTCAGCCCACACCGCGCCCTCGGAGGGCGGCGTGTACCAGTACAGCGTGGTGAAGCACTTCAGGCGCTCGGGCGGCCAGGACGGGCTCCTGCAGCTCATCAACGGCGTGTTGACTGTGACCGTGAACGACAGCGCCACCAATGCGGTGCTCGCCAGCGCCACCGTTGACCAGCTGCAGGGGTTTGCAATCGGTCAGAACACATGGAGCACCGAGGGTCTCGATCTGGTGCCCGCAGCGGAGGTG**CCGGAGGGGGTGCCCAAG**

**Bold**: Sequence corresponding to primers used to amplify the wild-type and mutant bands.

Blue Underline: Sequence corresponding to left *FAP70* 50-bp homology arm.

Blue Double Underline: Sequence corresponding to right *FAP70* 50-bp homology arm. Please note that the right primer used to amplify the bands is within the sequence corresponding to the right *FAP70* 50-bp homology arm.

Underline: gRNA recognition site.

Grey box: Protospacer adjacent motif (PAM).

Orange: Discrepancies between our *AphVIII* plasmid and the sequence from the Chlamydomonas Resource Center.
