# Supplementary material for: TIM, a targeted insertional mutagenesis method utilizing CRISPR/Cas9 in Chlamydomonas reinhardtii
Source: PLoS One. 2020 May 13;15(5):e0232594. doi: 10.1371/journal.pone.0232594 (PMC7219734; doi:10.1371/journal.pone.0232594)
Supplement: S2 Appendix — (DOCX) [file pone.0232594.s002.docx]

**Appendix S2. Alignment of sequences of donor DNA, wild-type DNA, and DNA from *fap70* mutants C6 and D6**

Donor DNA GCTCGCCAGCGCCACCGTTGACCAGCTGCAGGGGTTTGCAATCGGTCAGA 50

WT GCTCGCCAGCGCCACCGTTGACCAGCTGCAGGGGTTTGCAATCGGTCAGA 252

C6 GCTCGCCAGCGCCACCGTTGACCAGCTGCAGGGGTTTGCAATCGGTCAGA 252

D6 GCTCGCCAGCGCCACCGTTGACCAGCTGCAGGGGTTTGCAATCGGTCAGA 252

Donor DNA -------------------------------------------------- 50

WT ACACATGGAGCACCGAGGGTCTCGATCTGGTGC----------------- 285

C6 ACACATGGAGCACCGAGGGTCTCGAC------------------------ 278

D6 ACACATGGAGCACCGAGGGTTAATTTCGAGCTTGGCGTAATCATGGTCAT 302

Donor DNA -------------------------------------------------- 50

WT -------------------------------------------------- 285

C6 --------------------------------------------------

D6 AGCTGTTTCCTGTGTGAAATTGTTATCCGCTCACAATTCCACACAACATA 352

Donor DNA -------------------------------------------------- 50

WT -------------------------------------------------- 285

C6 --------------------------------------------------

D6 CGAGCCGGAAGCATAAAGTGTAAAGCCTGGGGTGCCTAATGAGTGAGCTA 402

Donor DNA -----------------------------GTAAAACGACGGCCAGTGAAT 71

WT -------------------------------------------------- 284

C6 --------------------------------------------------

D6 ACTCACATTAATTGCGTTGCGCTCACTGT--------------------- 431

Donor DNA TGTAATACGACTCACTATAGGGCGAATTGGAGCTCTTTCTTGCGCTATGA 121

WT -------------------------------------------------- 285

C6 --------------------------------------------------

D6 --------------------------------------------------

Donor DNA CACTTCCAGCAAAAGGTAGGGCGGGCTGCGAGACGGCTTCCCGGCGCTGC 171

WT -------------------------------------------------- 285

C6 --------------------------------------------------

D6 --------------------------------------------------

Donor DNA ATGCAACACCGATGATGCTTCGACCCCCCGAAGCTCCTTCGGGGCTGCAT 221

WT -------------------------------------------------- 285

C6 ----------------------------------------------GCAT 282

D6 ------------------------------------------GGCTGCAT 439

Above is an alignment of donor DNA, wild-type (WT) DNA, and DNA from *fap70* mutants C6 and D6 starting from the left homology arm at the 5’ end of the sequences. Sequences from the strains C1, C2, C3, D1, and D4 are not included in this alignment because they match perfectly with the donor DNA sequence, Compared with the donor DNA, C6 had a 26-bp insertion, which aligned with the wild-type sequence, except for the substitution of a C for a T at its 3’ end, followed by a 167-bp deletion. D6 had a 179-bp insertion followed by a 163-bp deletion. Within the 179-bp insertion in D6, the first 20 bps match the wild-type *FAP70* sequence and the next 158 bps match bps 1496-1653 of the donor DNA. Blue underlined sequence is the left 50-bp homology arm in the donor DNA. The PAM sequence in the wild-type sequence is shaded grey.

Donor DNA GTAATCATGG-------------------------TCATAGCTGTTTCCT 1536

WT --------------------GTCTCGATCTGGTGC--------------- 285

C6 GTAATCATGGGCGGAGCGACCCATGCATCTGGTGC--------------- 1607

Donor DNA GTGTGAAATTGTTATCCGCTCACAATTCCACACAACATACGAGCCGGAAG 1586

WT -------------------------------------------------- 285

C6 --------------------------------------------------

Donor DNA CATAAAGTGTAAAGCCTGGGGTGCCTAATGAGTGAGCTAACTCACATTAA 1636

WT -------------------------------------------------- 285

C6 --------------------------------------------------

Donor DNA TTGCGTTGCGCTCACTGCCCGCTTTCCAGTCGGGAAACCTGTCGTGCCAG 1686

WT -------------------------------------------------- 285

C6 --------------------------------------------------

Donor DNA CCGCAGCGGAGGTGCCGGAGGGGGTGCCCAAGGTGCGCGCGCCGGCCGCT 1736

WT CCGCAGCGGAGGTG**CCGGAGGGGGTGCCCAAG** 317

C6 CCGCAGCGGAGGTGCCGGAGGGGGTGCCCAAG 1639

Above is an alignment of donor DNA, wild-type (WT) DNA, and DNA from *fap70* mutant C6 at the 3’ end. The sequences from the other mutants are not included in this alignment because they match the donor DNA sequence perfectly in this region. Compared with the donor DNA, C6 had a 25-bp/165-bp insertion/deletion at the 3’ end of the paromomycin cassette immediately before the region corresponding to the donor DNA homology arm. The last 9 bps of the 25-bp insertion match the wild-type sequence beginning 3 bps upstream of the PAM sequence. Blue double underlined sequence is the right 50-bp homology arm in the donor DNA. Bold sequence in the wild-type sequence corresponds to the 3’-end primer used to amplify the mutation site in the mutants.
