# Supplementary material for: TIM, a targeted insertional mutagenesis method utilizing CRISPR/Cas9 in Chlamydomonas reinhardtii
Source: PLoS One. 2020 May 13;15(5):e0232594. doi: 10.1371/journal.pone.0232594 (PMC7219734; doi:10.1371/journal.pone.0232594)
Supplement: S1 Table — (DOCX) [file pone.0232594.s003.docx]

**Table S1. List of crRNA and primer sequences**

| Name | Sequence 5’-3’ | Application |
| --- | --- | --- |
| FAP70 crRNA | /AlTR1/rGrArGrCrArCrCrGrArGrGrGrUrCrUrCrGrArUrCrGrUrUrUrUrArGrArGrCrUrArUrGrCrU/AlTR2/ | crRNA for *FAP70* gene |
| IFT81 crRNA-1 | /AlTR1/rArCrArGrArArArGrUrGrGrArCrGrUrUrGrCrGrArGrUrUrUrUrArGrArGrCrUrArUrGrCrU/AlTR2/ | crRNA for *IFT81* gene designed using the IDT website |
| IFT81-crRNA-2 | /AlTR1/rCrArGrArCrGrGrCrGrGrArCrCrGrGrCrUrUrArUrGrUrUrUrUrArGrArGrCrUrArUrGrCrU/AlTR2/ | crRNA for *IFT81* gene designed using the CRISPR-direct website |
| MOT17 crRNA | /AltR1/rArArGrArGrCrGrGrArArCrGrArCrCrArCrArUrUrGrUrUrUrUrArGrArGrCrUrArUrGrCrU/AltR2/ | crRNA for *MOT17* gene |
| CDPK13 crRNA | /AltR1/rGrGrArGrArArGrArUrGrCrArCrUrGrUrCrUrGrCrGrUrUrUrUrArGrArGrCrUrArUrGrCrU/AltR2/ | crRNA for *CDPK13* gene |
| IFT43 crRNA | /AltR1/rGrArGrGrArCrArGrCrArArGrArArGrUrUrUrGrUrGrUrUrUrUrArGrArGrCrUrArUrGrCrU/AltR2/ | crRNA for *IFT43* gene |
| CEP131 crRNA-1 | /AltR1/rGrCrArGrGrCrGrUrUrGrArCrGrArUrGrCrGrCrUrGrUrUrUrUrArGrArGrCrUrArUrGrCrU/AltR2/ | crRNA for *CEP131* gene |
| CEP131 crRNA-2 | /AltR1/rGrArCrArCrCrUrGrGrCrCrUrUrCrGrUrGrGrArCrGrUrUrUrUrArGrArGrCrUrArUrGrCrU/AltR2/ | crRNA for *CEP131* gene |
| Paromomycin Forward (F) | GTAAAACGACGGCCAGTGAG | PCR primers to amplify paromomycin- resistance cassette |
| Paromomycin Reverse (R) | CTGGCACGACAGGTTTCCCG |  |
| FAP70 long arm paromomycin F | GCTCGCCAGCGCCACCGTTGACCAGCTGCAGGGGTTTGCAATCGGTCAGAGTAAAACGACGGCCAGTGAG | PCR primers to amplify FAP70 donor DNA with 50-bp homology arms and paromomycin resistance |
| FAP70 long arm paromomycin R | AGCGGCCGGCGCGCGCACCTTGGGCACCCCCTCCGGCACCTCCGCTGCGGCTGGCACGACAGGTTTCCCG |  |
| FAP70 short arm paromomycin F | CTGCAGGGGTTTGCAATCGGTCAGAGTAAAACGACGGCCAGTGAG | PCR primers to amplify FAP70 donor DNA with 25-bp homology arms and paromomycin resistance |
| FAP70 short arm paromomycin R | ACCCCCTCCGGCACCTCCGCTGCGGCTGGCACGACAGGTTTCCCG |  |
| IFT81-1 long arm paromomycin F | GCCCCTCTGCATGTGCTGGACATCTCGCTTACCGCTTCGCACGCTGACGCGTAAAACGACGGCCAGTGAG | PCR primers to amplify IFT81 donor DNA with 50-bp homology arms and paromomycin resistance. This donor DNA is paired with IFT81 gRNA-1 recognition site. |
| IFT81-1 long arm paromomycin R | CTTGATGATTTTCAGGAAGCCAATAAGCCGGTCCGCCGTCTGGTCCGGAACTGGCACGACAGGTTTCCCG |  |
| IFT81-2 long arm paromomycin F | CTTCGCACGCTGACGCTGCGCAAACAGAAAGTGGACGTTGCGAAGGAAGTGTAAAACGACGGCCAGTGAG | PCR primers to amplify IFT81 donor DNA with 50-bp homology arms and paromomycin resistance. This donor DNA is paired with IFT81 gRNA-2 recognition site. |
| IFT81-2 long arm paromomycin R | CTTGATGATTTTCAGGAAGCCAATAAGCCGGTCCGCCGTCTGGTCCGGAACTGGCACGACAGGTTTCCCG |  |
| MOT17 long arm paromomycin F | ACTACGCGCGGAAACGGTACCTCAAACAGGTCAAAGAGCGGAACGACCACGTAAAACGACGGCCAGTGAG | PCR primers to amplify MOT17 donor DNA with 50-bp homology arms and paromomycin resistance |
| MOT17 long arm paromomycin R | CGCTGCACGATGATGGCCCGCTCCGCCTCCAGGTTGAGCTCCGCCCGAATCTGGCACGACAGGTTTCCCG |  |
| CDPK13 long arm paromomycin F | GATGCAGGGGCACTTCAGCGAAAAGGACGCTTCGGAGAAGATGCACTGTCGTAAAACGACGGCCAGTGAG | PCR primers to amplify CDPK13 donor DNA with 50-bp homology arms and paromomycin resistance |
| CDPK13 long arm paromomycin R | GTCTCTGTGGATGATGTTCTTGGAGTGCGCGTACGCAATGAAGTCCAGCACTGGCACGACAGGTTTCCCG |  |
| Hygromycin F | GAATTCGATATCAAGCTTCTTTC | PCR primers to amplify hygromycin-resistance cassette |
| Hygromycin R | CGCTTCAAATACGCCCAG |  |
| FAP70 long arm hygromycin F | GCTCGCCAGCGCCACCGTTGACCAGCTGCAGGGGTTTGCAATCGGTCAGAGAATTCGATATCAAGCTTCTTTC | PCR primers to amplify FAP70 donor DNA with 50-bp homology arms and hygromycin resistance |
| FAP70 long arm hygromycin R | AGCGGCCGGCGCGCGCACCTTGGGCACCCCCTCCGGCACCTCCGCTGCGGCGCTTCAAATACGCCCAG |  |
| RACK1 | CAAGCTGAAGAACAACCTGGTG | PCR primers to amplify *RACK1* gene |
| RACK1 | CTTGCTGGTGATGTTGAACTCG |  |
| FUS1 F | TCCAACGCATAGCCATCAAC | PCR primers to amplify *FUS1* gene |
| FUS1 R | TGTTTGCTAGGGGTGCAATG |  |
| FAP70 F | CGCAGCGCGAAACCCACATC | PCR primers to genotype *FAP70* gene |
| FAP70 R | CTTGGGCACCCCCTCCGG |  |
| IFT81 F | GCTCCTTCATTTATCCGGACCC | PCR primers to genotype *IFT81* gene |
| IFT81 R | CAGCTGGCGGAAGAGCAG |  |
| MOT17 F | GTTCTGAGCTTGAGCGAGGT | PCR primers to genotype *MOT17* gene |
| MOT17 R | CTTCCACTAGCACTGCAACG |  |
| CDPK13 F | CTGGGGTGGGGTATGAGTGT | PCR primers to genotype *CDPK13* gene |
| CDPK13 R | CTCTGGCGCCACGTAGTATG |  |
| IFT43 F | ACAACGCTGTCCCCTTATTG | PCR primers to genotype *IFT43* gene |
| IFT43 R | AGGTTCTTGCGGCTGACTT |  |
| CEP131-1 | CATGCACGCATACACCACAG | PCR primers to genotype *CEP131* gene for gRNA-2 |
| CEP131-2 | ACACTTCCCACCCATTCACC |  |
| CEP131-3 | CCCACTTTCCTGCGAAGAAG | PCR primers to genotype *CEP131* gene for gRNA-1 |
| CEP131-4 | GGGCATGGCCTCATAAAGAC |  |
| AphVIII-1 | GTGGGATGGGGCGGTATC | Sequencing primer for paromomycin cassette |

For the *IFT43 and CEP131* genes, the paromomycin cassette without homology arms was used as donor DNA.
