# Supplementary material for: TIM, a targeted insertional mutagenesis method utilizing CRISPR/Cas9 in Chlamydomonas reinhardtii
Source: PLoS One. 2020 May 13;15(5):e0232594. doi: 10.1371/journal.pone.0232594 (PMC7219734; doi:10.1371/journal.pone.0232594)
Supplement: S2 Table — (DOCX) [file pone.0232594.s004.docx]

| Protein Name | JGI Transcript Name | NCBI Gene ID |
| --- | --- | --- |
| FAP70 | Cre07.g345400.t1.2 | 5718018 |
| IFT81 | Cre17.g723600.t1.2 | 5722735 |
| FAP305/MOT17 | Cre11.g482300.t1.2 | 5722956 |
| CDPK13^1^ | Cre13.g571700.t1.1 | 5719151 |
| IFT43/MOT41 | Cre06.g251200.t1.2 | 5722198 |
| MOT3/CEP131 | Cre09.g394732.t1.1 | 5720302 |
| RACK1^2^ | Cre06.g278222.t1.1 | 5723548 |
| FUS1 | Cre06.g252750.t1.1 | 5722167 |

**S2 Table. Gene Identifications**

^1^Calcium-dependent protein kinase 13

^2^Receptor of activated protein kinase C1
