# Supplementary material for: TIM, a targeted insertional mutagenesis method utilizing CRISPR/Cas9 in Chlamydomonas reinhardtii
Source: PLoS One. 2020 May 13;15(5):e0232594. doi: 10.1371/journal.pone.0232594 (PMC7219734; doi:10.1371/journal.pone.0232594)
Supplement: S1 Fig — (PDF) [file pone.0232594.s005.pdf]

Fig 1A

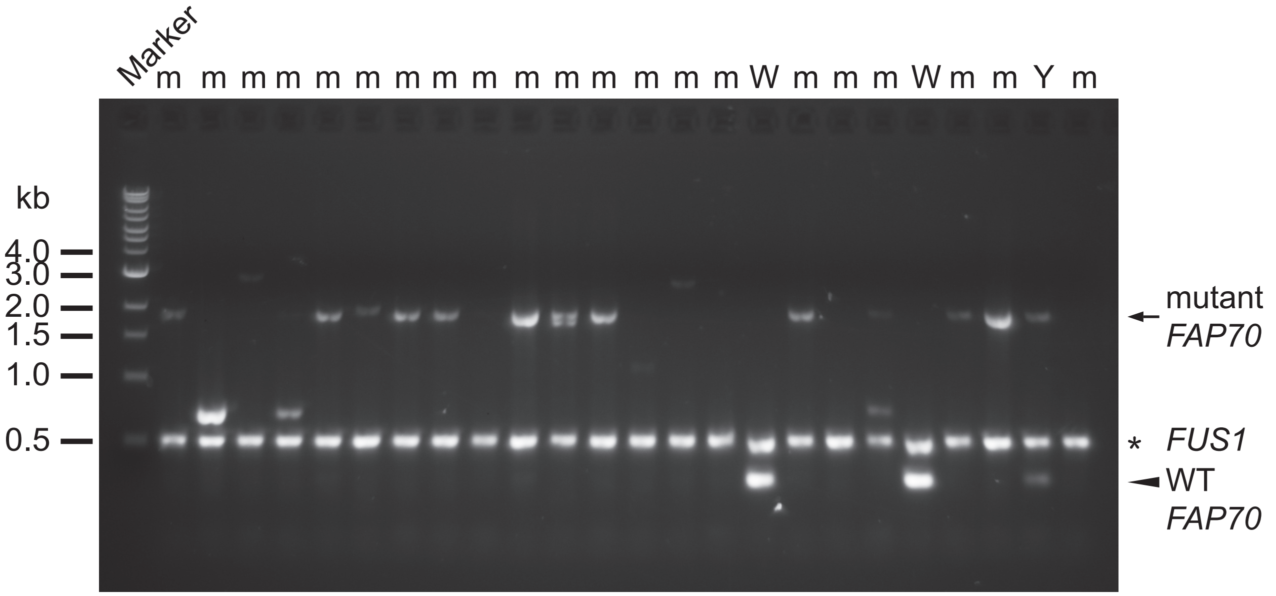

Marker: 1 kb Extend DNA Ladder (NEB #N3239S); m: *fap70* mutant; W: *FAP70* wild type; Y: mixed *fap70* mutant and *FAP70* wild-type colonies

Fig 1C upper panel

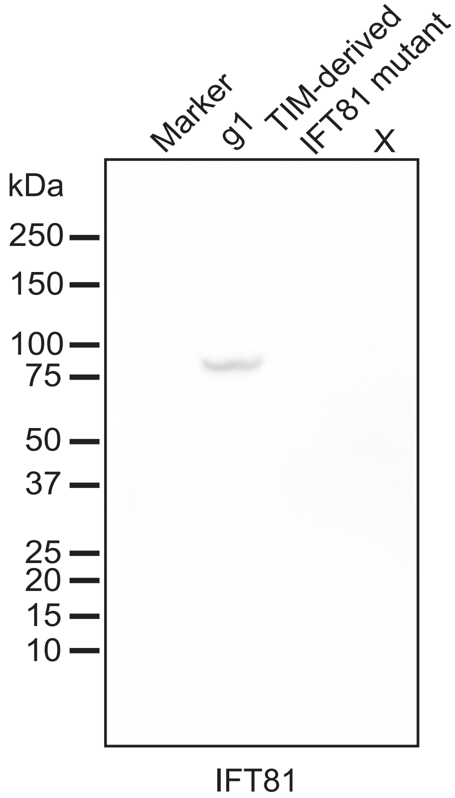

Fig 1C lower panel

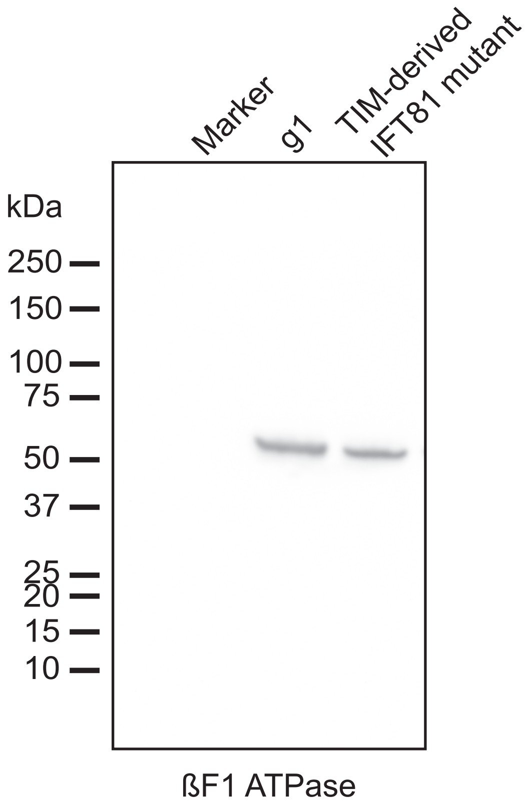

Marker: Bio-Rad Precision Plus Protein Standards (#161-0373)
